# CO_2_ Electroreduction to Formate: Advancing toward Scalable Technologies

**DOI:** 10.1021/acsaem.5c03659

**Published:** 2026-02-06

**Authors:** Jose Antonio Abarca, Guillermo Díaz-Sainz, Ángel Irabien

**Affiliations:** Departamento de Ingenierías Química y Biomolecular, 16761 Universidad de Cantabria, Avenida de los Castros s/n, 39005 Santander, Spain

**Keywords:** CO_2_ electroreduction, Formate production, Scale-up challenges, GDE, MEA, Electrolyzer engineering

## Abstract

Scaling up CO_2_ electroreduction to formate requires optimizing electrode design and reactor configuration. Gas diffusion electrodes and membrane electrode assemblies enable high CO_2_ transport and production rates, but long-term stability remains challenging. Flow cells offer better scalability than H-type cells, supporting continuous operation and improved mass transfer. In large systems, uniform CO_2_ distribution and pressure balance are critical to prevent performance losses. Strategies like stacked cell designs to increase electrolyzer surface area must also be considered. Addressing electrode durability and reactor engineering challenges is essential for advancing industrial implementation of CO_2_ electroreduction to formate.

## Graphical abstract

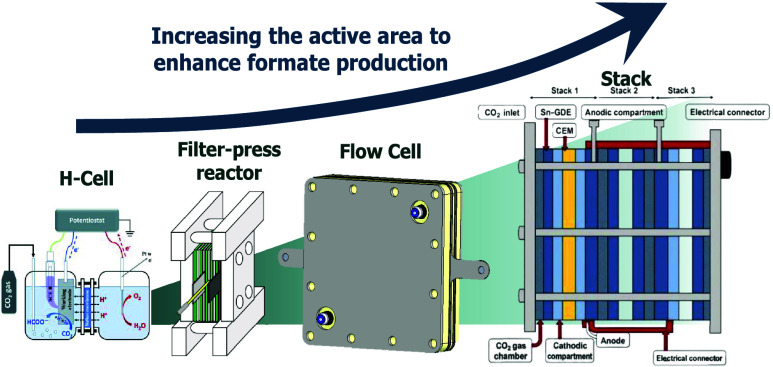

Human activity and the combustion of fossil fuels have led to a continuous increase in CO_2_ and other greenhouse gas emissions, surpassing the planet’s natural capacity for absorption. As a result, atmospheric CO_2_ concentrations have risen sharply, reaching a threshold of 425 ppm in October 2025.^1^ In response, various international organizations, led by the United Nations (UN), have promoted agreements and treaties aimed at the reduction of CO_2_ emissions.^2^ One of the most significant milestones is the adoption of the Sustainable Development Goals, with particular emphasis on SDG 7 (Affordable and Clean Energy) and SDG 13 (Climate Action), which serve as key pillars in the global effort to reduce CO_2_ emissions.^3^ At the European level, the European Commission has established a long-term objective of making Europe the first net-zero emission continent by 2050.^4^

Among the most widely developed strategies to reduce CO_2_ emissions are the adoption of renewable energy sources, improvements in energy efficiency, and the promotion of circular economy principles.^5^ However, achieving climate neutrality requires additional efforts in hard-to-abate sectors, where CO_2_ emissions are inherently tied to industrial processes and cannot be eliminated through conventional approaches.^6^ Within the portfolio of decarbonization strategies applicable to these challenging sectors, Carbon Capture, Utilization, and Storage (CCUS) technologies have gained increasing relevance.^7^ These technologies begin with the capture of CO_2_ from point sources using techniques such as amine absorption, adsorption, or membrane separation.^8^ Once captured, CO_2_ can follow two main pathways: storage in geological formations, such as depleted oil and gas reservoirs,^9^ or utilization as a feedstock or chemical reagent. The latter option offers additional advantages from a circular economy perspective by valorizing a waste product.^10^ CO_2_ can be used directly in applications such as agriculture or converted into value-added products through various conversion routes, including thermochemical, electrochemical, biochemical, photocatalytic, or mineralization processes.^11^

Among these conversion pathways, electrochemical reduction (electroreduction) of CO_2_ to value-added products stands out as a particularly promising approach. This process involves applying a potential difference between two electrodes, providing the electrons needed to activate and transform the CO_2_ molecule.^12^ One of the main environmental and economic advantages of this technology is its capacity to store renewable energy in the form of chemical bonds.^13^ The electrochemical system typically consists of two electrodes: the cathode (working electrode), where CO_2_ reduction occurs, and the anode (counter electrode), where a coupled oxidation reaction takes place, most commonly the oxygen evolution reaction (OER).^14^ To separate both compartments while maintaining ionic conductivity, an ion-exchange membrane is placed between the electrodes.^15^

A wide range of reduction products can be generated from CO_2_, as illustrated in Figure 1. The product distribution depends on several factors, including the number of electrons transferred, the catalyst used, and the reaction medium.^17^ Among the most relevant products are carbon monoxide (CO), formate or formic acid (HCOO^–^/HCOOH), ethylene (C_2_H_4_), methane (CH_4_), methanol (CH_3_OH), and ethanol (C_2_H_5_OH).^18^ Each of these reduction reactions occurs at different applied potentials at the working electrode.^19^ A major competing reaction at the cathode is the hydrogen evolution reaction (HER), which occurs during water reduction. This reaction occurs within a potential range similar to that of many CO_2_ reduction reactions, leading to competition for electron supply and thus reducing the Faradaic efficiency toward the desired CO_2_-derived products.^20^

**Figure 1:**
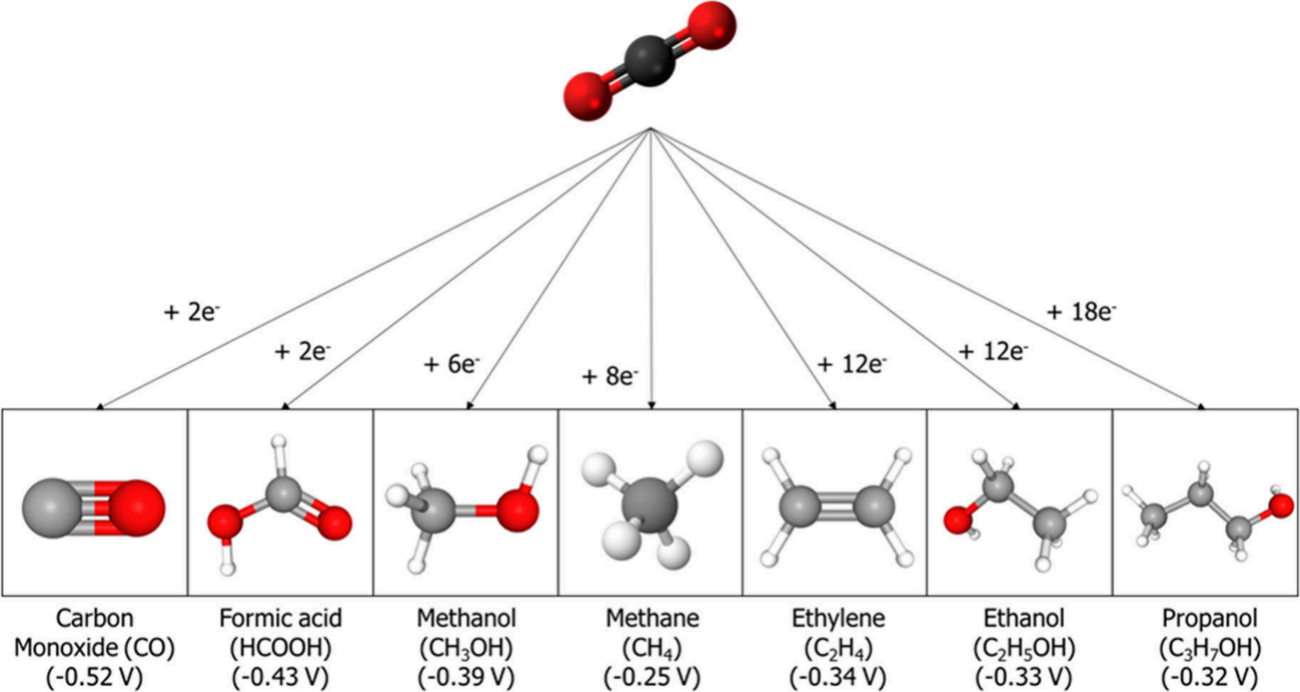
Diagram of the different CO_2_ reaction products as a function of the number of electrons transferred. Adapted with permission from Leonzio et al.^16^ Copyright 2024 Elsevier.

Formate and formic acid are two key products of CO_2_ reduction with significant industrial relevance.^21^ They serve as precursors for high-value products and raw materials. Common industrial applications include silage preservation, animal feed additives, textile finishing, antifreeze agents, and intermediates in the chemical and pharmaceutical industries.^22,23^ Formic acid is particularly attractive due to its unique versatility. Global demand for HCOOH is expected to rise substantially, from 750,000 tonnes in 2021 to 1,300,000 tonnes by 2035, driven by technological progress.^24^ Emerging applications include its use as reactant in direct formic acid fuel cells and as a hydrogen storage medium, thanks to its high hydrogen density and liquid state, which facilitate storage and transport. Currently, global formic acid production is largely based on fossil fuel-derived processes, such as the hydrolysis of methyl formate.^25^ Consequently, it is essential to explore alternative production routes, such as CO_2_ electroreduction, which offer a more sustainable approach by leveraging renewable energy and promoting circular-economy principles to transform CO_2_, an industrial waste, into valuable products. In this context, the industrial scaling process should ultimately aim to integrate the electrocatalytic conversion of CO_2_ to formate within the industrial production workflow, alongside CO_2_ capture. This represents a new paradigm of complete CO_2_ recycling within the industrial plant itself, as shown in Figure 2. The formate/formic acid could serve either as a molecule for renewable energy storage or as a reagent within the industrial process.

**Figure 2:**
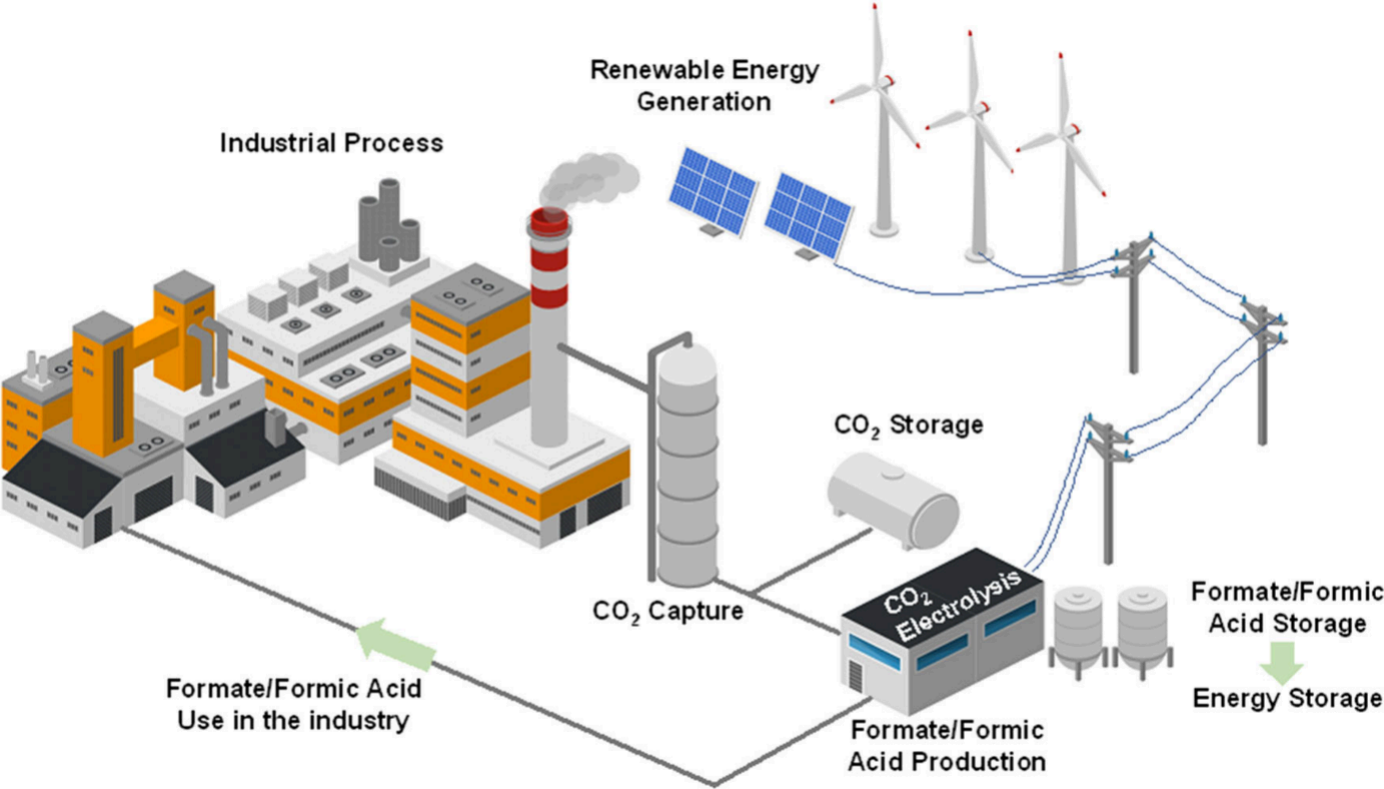
Schematic illustration of an industrial plant implementing CO_2_ electrolysis as part of the global in situ CO_2_ recycling system within the industry.

Interest in this technology has expanded rapidly in recent years due to its techno-economic and environmental advantages. Numerous studies have focused on the optimization of the CO_2_ electroreduction process to formate, exploring various catalytic materials, particularly Bi, Sn, or In (Figure 3), along with the system parameter optimization and electrode composition. These efforts have yielded promising laboratory-scale results, achieving Faradaic efficiencies above 90% and formate concentrations exceeding 300 g L^–1^. Such achievements have paved the way for the scaling-up of the technology, with two main objectives: (i) expanding the active area of the CO_2_-to-formate electrolyzers to process larger CO_2_ volumes in a single pass; and (ii) boosting formate production capacity to compete with conventional routes and enable industrial implementation of this technology.

**Figure 3:**
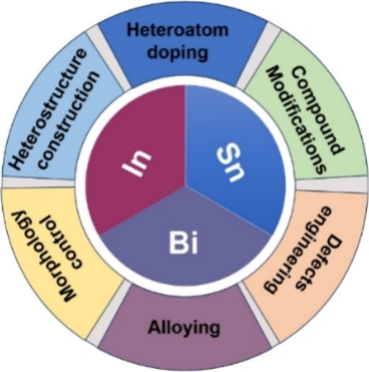
Summary of the strategies used to optimize Sn-, In-, and Bi-based electrocatalysts for the conversion of CO_2_ to formate or formic acid. Reproduced with permission from Liu et al.^26^ Copyright 2023 Wiley.

However, scaling up from laboratory devices, with active areas of only a few cm^2^ and CO_2_ flow rates of a few mL min^–1^, to pilot or industrial-scale electrolyzers, with active areas in the hundreds or thousands of cm^2^ capable of processing large CO_2_ streams, introduces multiple challenges across the electrochemical system configuration.

The first key aspect is electrode preparation, particularly the working electrode or cathode, which plays a decisive role in achieving efficient CO_2_ conversion. A wide variety of electrode configurations are used at the cathode or working electrode, including flat plates, thin film electrodes, and catalyst-coated membranes.^27^ However, gas diffusion electrodes (GDEs) and membrane electrode assemblies (MEAs) are among the most suitable configurations for scale-up, as their structures allow the processing of gaseous CO_2_ streams, reducing mass transfer resistance and enhancing productivity toward formate.^28,29^ On the one hand, GDEs enable the diffusion of gaseous CO_2_ through the electrode structure to reach the catalytic surface. Typically, a GDE consists of a conductive, macroporous, and hydrophobic carbon-based substrate connected to the power source, onto which the catalyst layer (CL) is deposited. A microporous layer (MPL), composed of carbon materials and polymers, may be placed between the substrate and the catalyst to enhance CO_2_ mass transport. This electrode configuration promotes efficient three-phase contact between the gas, liquid electrolyte, and solid catalyst, offering key advantages such as maintaining high CO_2_ concentrations at the reaction interface and enabling operation at high current densities.^30,31^ On the other hand, an MEA features a sandwich-like structure composed of three functional layers.^32^ At its core lies an ion-exchange membrane, typically a cation exchange membrane, which enables proton transport from the anode to the cathode. The cathode and the anode are placed in direct contact with the membrane to minimize mass and energy transfer resistances.^33^ This integrated configuration is essential for achieving efficient electrochemical performance and high product selectivity.

The manufacturing capability of such electrodes is critical; fabrication techniques must allow reproducible and, ideally, automated production of large area electrodes.^27^ Standardizing these fabrication processes is essential for industrial deployment. Deposition techniques such as spray coating, sputtering, or electrodeposition are particularly advantageous, as they enable the uniform application of catalysts onto substrates in a scalable and reproducible manner, facilitating the transition from lab- to industrial-scale production of high-surface-area electrodes.

Beyond electrode preparation, additional challenges emerge concerning electrode stability and GDE deactivation phenomena. Two of the most critical issues are salt precipitation and electrode flooding (Figure 4). Salt precipitation, Figure 4.a, primarily occurs under alkaline conditions, where CO_2_ reacts with hydroxide ions to form bicarbonate and carbonate species.^21^ In the presence of high concentrations of metal cations (e.g., K^+^, Na^+^, Cs^+^), these anions can lead to the formation of poorly soluble salts that accumulate within the GDE structure.^34^ This precipitation process is exacerbated in gas-phase operation, where no liquid catholyte is present,^35^ resulting in blockage of the electrode’s porous structure. Consequently, CO_2_ transport to the catalytic layer is hindered, reducing access to active sites and promoting secondary reactions such as the HER.^36^ This significantly lowers the Faradaic efficiency toward formate and represents a rapid deactivation mechanism, often occurring within the first few hours of GDE operation.

**Figure 4:**
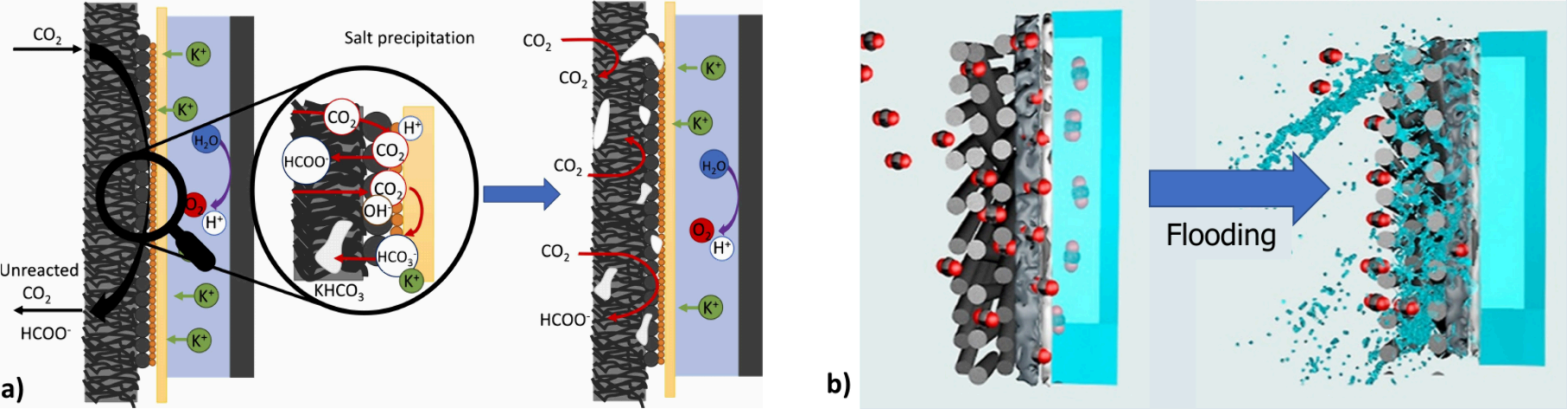
Schematic representation of the a) Salt precipitation and b) GDE flooding phenomena. Adapted with permission from Abarca et al. and Wu et al.^37,40^ Copyright 2024 Elsevier. Copyright 2022 Elsevier.

To address salt precipitation in zero-gap configurations, several mitigation strategies have been proposed. Some focus on modifying the anolyte composition, using acidic solutions that effectively reduce salt formation in the cathode GDE.^37^ Other studies emphasize the beneficial effect of water injection into the cathode compartment, alongside the CO_2_ stream, to dissolve accumulated salts and products.^38,39^ Additionally, membrane properties, such as type, thickness, and permeability, play a crucial role in water management within the cathode GDE, directly influencing salt precipitation and accumulation.^38^

Flooding in the GDE represents another major challenge that undermines the long-term stability of CO_2_ electroreduction systems. Unlike salt precipitation, flooding tends to develop gradually. During operation, the hydrophobic properties of the GDE and its interaction with the electrolyte evolve, allowing the catholyte to infiltrate the electrode structure, as seen in Figure 4.b. This penetration can obstruct the porous gas diffusion layer (GDL), limiting CO_2_ transport to the catalyst layer (CL) and ultimately reducing the electrode’s efficiency.^41,42^

This phenomenon has been widely studied, and various mitigation strategies have been proposed.^42–47^ One common approach involves controlling the pressure gradient between the CO_2_ inlet and the catholyte side. By using the GDE as a selective barrier, this pressure control effectively limits liquid intrusion.^48^ Material design also plays an essential role: enhancing the hydrophobicity and tuning the wettability of the GDE helps maintain the triple-phase boundary, preventing undesired liquid penetration.^45,47^ Alternatively, modifying the GDE microstructure to promote efficient drainage of infiltrated catholyte has also shown promise in reducing flooding and preserving long-term performance.^49^

The phenomena mentioned earlier occur on a macroscopic scale and pose challenges to the operation of the ERCO_2_ system from a scaling perspective. However, these macroscopic phenomena are driven by processes taking place at a microscopic scale within the pores of the GDE, where the behavior of the CO_2_ reduction reaction and its local microenvironment play a critical role in overall system performance.^50^ First, the local hydrophobicity of the catalyst can significantly influence the interface where CO_2_ reduction occurs. A moderate level of hydrophobicity can create a microenvironment that balances gaseous CO_2_ and liquid electrolyte within the catalyst layer, reducing the diffusion layer thickness, accelerating CO_2_ mass transport, and forming highly active reaction zones near solid–liquid–gas interfaces. This enables high-rate CO_2_ electrolysis to formate.^51^ Additionally, the local pH also plays a fundamental role in reaction selectivity, particularly in suppressing the HER. Maintaining favorable local pH conditions near the catalyst pores promotes CO_2_ conversion. However, shifts in local pH, caused by factors such as changes in the concentration of positive species or proton donors, can impact performance. An excessively high pH can reduce the available CO_2_ concentration by forming (bi)­carbonate ions, which may precipitate as salts when combined with cations present in the reaction zone.^52^ Therefore, the liquid electrolyte, or cation migration through the membrane in the case of zero-gap configurations, must maintain an optimal local pH window to enable stable CO_2_ electroreduction to formate over extended operation periods. Electrostatic forces and the resulting electrical double layer (EDL) also have a significant influence on the microscopic phenomena described above. At higher applied potentials, the concentration of cations increases within the EDL region.^53^ Inside the catalyst pores, a local pH drop may occur because the strong electric field formed in the EDL can attract not only cations (K^+^, Na^+^) from the electrolyte solution but also H^+^ ions.^54^ This solvent polarization within the EDL critically affects the kinetics of CO_2_ electroreduction, influencing both the local pH and the proton-donor concentration, as well as the long-term stability of the system.

Electrode stability remains a critical bottleneck for the industrial-scale implementation of CO_2_ electroreduction technologies. To ensure technical, economic, and environmental competitiveness, research must focus on extending the effective operational lifetime of the electrodes while maintaining productivity and selectivity. Current operational periods, typically limited to a few days, must be expanded to weeks or longer, improving system reliability and maximizing the utilization of scarce resources such as the catalyst materials.

Another key factor for successful scale-up is the design of the CO_2_ electroreduction.^55^ While H-type cells are commonly used in early stage studies due to their simplicity, they suffer from low reaction rates and highly diluted products.^56^ In contrast, flow cells offer a scalable alternative, enabling continuous CO_2_ gas feed and enhanced mass transfer at the catalyst surface.^57,58^ These systems operate at the gas–liquid–solid triple-phase boundary using GDEs, enhancing efficiency.

Flow cell configurations for CO_2_ electroreduction to formate can operate under liquid-phase or gas-phase configurations. In the liquid-phase setup, a liquid catholyte is present between the GDE and the ion-exchange membrane, offering greater system stability but producing more diluted formate.^59^ In contrast, in gas-phase operation, humidified CO_2_ is fed directly to the GDE in close contact with the membrane, eliminating the catholyte and enabling formate concentrations exceeding 300 g L^–1^ at lab scale.^35^ However, this approach is more susceptible to salt precipitation in the GDE, which limits long-term operation. Thus, the choice between these configurations involves a trade-off between product concentration and operational robustness.

Scaling up CO_2_ electrolyzer systems introduces multiple engineering challenges in both design and operation. While laboratory-scale devices typically feature electrode areas of only cm^2^, industrially relevant prototypes require areas of at least 100 cm^2^ for meaningful performance validation under realistic conditions. However, this scale-up introduces engineering challenges often overlooked at smaller scales.^60^ Key issues include the design and fabrication of large-area electrodes, uniform CO_2_ distribution across the electrode surface, and pressure management within the reactor. Ensuring uniform flow prevents mass transport limitations such as concentration gradients, dead zones, or high-velocity region.^61,62^

Beyond flow uniformity, pressure balancing across the GDE becomes increasingly important as system scale increases. Although often neglected at lab scale, pressure gradients can significantly affect performance and stability. Fink et al.^63^ illustrated potential failure scenarios: insufficient CO_2_ pressure relative to the liquid side can lead to GDE flooding, while excessive pressure can cause oversaturation, bubble formation, or carbonate losses.^48^ Therefore, maintaining a well-controlled pressure differential is essential for stable and efficient operation.

Despite recent progress, several challenges still hinder the scale-up of CO_2_ electroreduction systems. These include low CO_2_ conversion efficiencies, HER competition, high energy requirements, mass transport limitations, and the lack of scalable and efficient reactor components and architectures.^64^ Nevertheless, recent research shows encouraging advances toward overcoming these barriers.^65^

Only a few studies have specifically addressed the large scale-up of CO_2_ electroreduction to formate. For instance, Abarca et al.^66^ designed a 100 cm^2^ gas-phase electrolyzer (Figure 5.a), achieving promising up to 760 g L^–1^ and Faradaic Efficiencies of 67% under optimal operating conditions. Similarly, Izadi et al.^67^ investigated scale-up strategies for CO_2_-to-formate electrolyzer configurations using a liquid-phase configuration, comparing systems ranging from 10 cm^2^ to 100, 300, and 400 cm^2^. Their results showed that single-cell configuration (100 and 400 cm^2^) achieved comparable production rates (∼30 mM h^–1^) and efficiencies (∼80%), whereas the three-stack (300 cm^2^) exhibited lower efficiency (∼50%) due to increased HER activity and system complexity (Figure 5.b).

**Figure 5:**
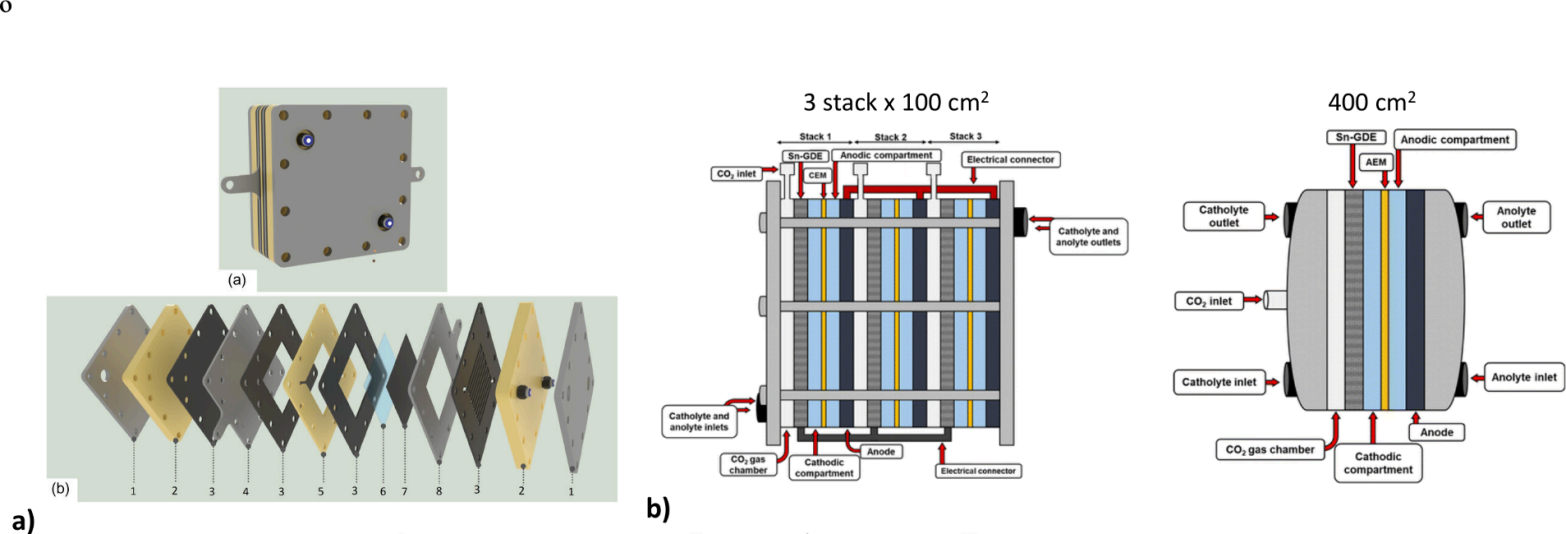
Schematic of the different CO_2_ electrolyzer configurations reported in literature for: a) 100 cm^2^ gas-phase electrolyzer, and b) 300 cm^2^ stack and 400 cm^2^ single electrode. Adapted with permission from Abarca et al., and Izadi et al.^63,66^ Copyright 2025 Wiley. Copyright 2026 Royal Society of Chemistry.

One of the largest active electrode areas reported to date was developed by Fink et al.,^63^ who constructed a 1526 cm^2^ single electrode and assembled a two-electrode stack reaching a total area of 3052 cm^2^. This system achieved a production rate of 0.5 kg of potassium formate per hour. Despite these high efficiencies, formate concentrations in liquid-phase configurations^63,67^ remained relatively low, typically between 3 and 9 g L^–1^.

As shown, several recent studies are beginning to address the fundamental challenges of scaling, focusing primarily on electrode fabrication and electrolyzer design. These efforts demonstrate the technical feasibility of scaling up the technology. However, further work is needed to optimize both the electrode configuration and the CO_2_-to-formate electrolyzer systems to maximize performance and enable competitiveness with conventional methods of formate/formic acid production. In this context, two key areas of action must be established. The first concerns the electrode itself: it is essential to determine the maximum durability of GDEs/MEAs to define the operational limits of this cathode configuration and assess its suitability for industrial-scale deployment. If these materials prove insufficiently robust, alternative electrode configurations must be explored, ones capable of maintaining high efficiency over extended operation periods. In this regard, metallic foams are emerging as a promising alternative to carbon-based cathodes.^68,69^

The second area involves scaling strategies for the electrolyzer. These can focus either on increasing the active area of individual electrodes or on designing stack configurations. For the latter, challenges related to the management of gas and liquid flows within the system must be addressed. Additionally, enabling gas-phase operation could offer significant industrial advantages due to the higher product concentrations achievable under such conditions. Furthermore, it is essential to increase the single-pass conversion efficiency, namely, the overall capacity to convert CO_2_ into the target product, in this case, formate. Achieving a high conversion rate of the CO_2_ fed into the electrolyzer will significantly enhance the attractiveness of this technology as a viable decarbonization strategy. In this regard, the impact of low CO_2_ concentrations and the presence of impurities must be thoroughly evaluated, as the effective integration of CO_2_ capture and conversion stages is critical for technically feasible scale-up. Ensuring compatibility and robustness under realistic feed conditions will be key to the industrial deployment of these systems.

For efficient scale-up and the implementation of the most suitable system for CO_2_ electroreduction to formate, tailored to the specific needs of each industrial facility, prior modeling and optimization of the systems are essential before their design and deployment. This approach enables the identification of optimal configurations and operating conditions, ensuring that the selected technology meets performance, cost, and integration requirements at an industrial scale. For this purpose, the use of data science and machine learning (ML) has emerged as a powerful tool to enhance the scaling and design of chemical processes by enabling the optimization of complex multivariable systems, such as the electrochemical reduction of CO_2_ to formate. In this context, ML facilitates the optimization of various operational parameters of the electrochemical system through the analysis of large volumes of experimental data, where relationships between variables are often nonlinear. Parameters such as applied potential, catalyst loading, CO_2_ flow rate, or reactor configuration can be correlated with key performance indicators, such as Faradaic efficiency, current density, or CO_2_ conversion, significantly reducing the number of experiments required to reach optimal conditions.^70^ Moreover, data-driven empirical modeling proves especially useful during the process scale-up stages, where the use of rigorous physical-mathematical models, such as computational fluid dynamics (CFD), may involve high computational costs.^71^ ML-based algorithms can complement or even replace these traditional models, enabling real-time design and control of complex flow cells.

So far, the main reported applications of ML in CO_2_ electroreduction to formate have focused on catalyst screening.^72^ However, one of the most promising areas of application is the analysis of degradation and deactivation phenomena in electrochemical systems. Through time-series analysis and anomaly detection, it is possible to identify abnormal operating patterns that correlate with known degradation mechanisms,^73^ such as GDE electrode flooding or salt precipitation. Applying ML to this type of analysis can significantly reduce the number of experiments required to identify electrode degradation mechanisms and enable predictions of their behavior under specific manufacturing and operating conditions. This capability is particularly valuable during prototype validation and pilot-scale testing, where long-term operational stability must be ensured.

Once electrode scaling is achieved, with robust performance and high conversion efficiency, and the electrolyzer design is optimized, additional challenges may arise. Chief among them is energy demand. Powering the electrochemical conversion system with renewable energy sources significantly reduces the carbon footprint of formate production, while also serving as a strategy for energy storage in the form of chemical bonds. However, excessively high cell overpotentials can render the electroconversion process noncompetitive at industrial or larger scales. Therefore, minimizing energy losses and improving overall energy efficiency are critical to ensuring the viability of this technology for large-scale deployment. In recent years, several strategies have emerged to reduce energy requirements and enhance the overall energy efficiency of the system. One such approach involves coupling photoactive surfaces to enable both CO_2_ electroreduction and the associated oxidation reaction under solar or UV light irradiation. This setup generates an additional electron flow due to photon-induced excitation of photoactive materials, thereby reducing external energy dependence.^27^ Another promising strategy is the coupling of oxidation reactions with lower overpotentials than the OER, such as the oxidation of glycerol or ethanol.^74^ These alternatives have been proposed as effective means to enhance energy efficiency. Additionally, the development of alternative anodes with very high active surface areas, enabled by the use of metallic foams, has also contributed to significant reductions in energy demand.^75^ Although these strategies are still in early stages of development and limited to small-scale systems, they hold great potential for improving the overall competitiveness of CO_2_ electroreduction to formate, especially once they can be implemented at larger scales.

With all this in mind, and to establish a clear framework in which advances in scaling CO_2_ electroreduction to formate can bring the technology closer to industrial deployment, it is essential to define a set of key performance indicators (KPIs) to ensure that a sufficient technological maturity is achieved for industrial implementation. These KPIs (Table 1) focus on the reactor area and may vary depending on the required productivity, industrially relevant current density, selectivity toward the desired product, electrode stability, and favorable energy efficiency. Meeting these criteria ensures that the system is both technically feasible and economically attractive for large-scale formate production through CO_2_ electrolysis.

**Table 1:** Main Target Values for the Most Relevant KPIs for the Scaling up of CO_2_ Electroreduction to Formate toward Industrial Implementation^23,76,77^

KPI	Target Value
Electrode Area	>1000 cm^2^
Current density	>200 mA cm^–2^
Faradaic Efficiency toward formate	>90%
Electrode stability	>8000 h
Cell Voltage	<3.5 V
Energy Consumption	<500 kWh kmol^–1^
Energy Efficiency	40–60%
Single Pass Conversion Efficiency	>50%
Product Concentration	>20% wt.
	>85% wt. (If a purification stage is not considered)

Altogether, formate, together with CO, represents one of the most promising products for large-scale production via CO_2_ electroreduction, owing to the high yields and Faradaic efficiencies reported to date. Achieving commercial viability, however, requires addressing several key challenges, including the choice of electrode materials, fabrication methods, and long-term operational stability, and the design and construction of scalable electrolyzer systems. Despite these challenges, recent studies indicate that the short- to medium-term prospects for scaling this technology are highly promising. This technical feasibility, coupled with the growing demand for formate and formic acid as valuable chemical products, highlights the critical need for continued research focused on advancing the scalability and industrial implementation of CO_2_ electroreduction technologies.
